# The Prognostic Value of Androgen Receptor Splice Variant 7 in Castration-Resistant Prostate Cancer Treated With Novel Hormonal Therapy or Chemotherapy: A Systematic Review and Meta-analysis

**DOI:** 10.3389/fonc.2020.572590

**Published:** 2020-11-30

**Authors:** Zhize Wang, Haixiang Shen, Nieying Ma, Qinchen Li, Yeqing Mao, Chaojun Wang, Liping Xie

**Affiliations:** ^1^ Department of Urology, The First Affiliated Hospital, Zhejiang University School of Medicine, Hangzhou, China; ^2^ Key laboratory of Reproductive Genetic (Ministry of Education) and Department of Reproductive Endocrinology, Women’s Hospital, Zhejiang University School of Medicine, Hangzhou, China

**Keywords:** androgen receptor splicing variant 7, novel hormonal therapy, chemotherapy, prostate cancer, predictor, survival

## Abstract

**Purpose:**

This study aimed to evaluate the prognostic role of AR-V7 in terms of prostate-specific antigen (PSA) response, progression-free survival (PFS), and overall survival (OS) in CRPC patients treated with novel hormonal therapy (NHT) (Abiraterone and Enzalutamide) or taxane-based chemotherapy (Docetaxel and Cabazitaxel).

**Methods:**

A comprehensive literature search was conducted on PubMed, Embase, and the Web of Science from inception to February 2020. Studies focusing on the prognostic values of AR-V7 in CRPC patients treated with NHT or chemotherapy were included in our meta-analysis. The OS and PFS were analyzed based on Hazard ratios (HRs) and 95% confidence intervals (CIs). Furthermore, Odds ratios (ORs) and 95% CIs were summarized for the AR-V7 conversion after treatment and the PSA response.

**Results:**

The AR-V7 positive proportion increased significantly after NHT treatment (OR 2.56, 95% CI 1.51–4.32, P<0.001), however, it declined after chemotherapy (OR 0.51, 95% CI 0.28–0.93, P=0.003). AR-V7-positive patients showed a significantly decreased PSA response rate after NHT (OR 0.13, 95% CI 0.09–0.19, P<0.001) but not statistically significant for chemotherapy (OR 0.63, 95% CI 0.40-1.01, P=0.06). Notably, PFS (HR 3.56, 95% CI 2.53–5.01, P<0.001) and OS (HR 4.47, 95% CI 3.03–6.59, P<0.001) were worse in AR-V7-positive ttreated with NHT. Similarly, AR-V7 positivity correlated with poor prognosis after chemotherapy as evidenced by shorter OS (HR 1.98, 95% CI 1.48-2.66, P<0.001) and a significantly shorter PFS (HR 1.35, 95% CI 0.97-1.87, P=0.07).

**Conclusion:**

NHT treatment increased AR-V7 positive proportion whereas chemotherapy decreased it. Moreover, AR-V7 positivity correlated with lower PSA response, poorer PFS, and OS in CRPC treated with NHT, and shorter OS in patients receiving chemotherapy.

## Introduction

Taxane-based chemotherapy (Docetaxel and Cabazitaxel) and novel hormonal therapy (NHT) such as Enzalutamide and Abiraterone have been proven to improve overall survival (OS) and possess favorable safety profiles in metastatic castration-resistant prostate cancer (mCRPC) (1, 2). The prognosis of most mCRPC patients is poor which calls for development of effective biomarkers to predict treatment outcomes and choices (3, 4). The constitutively active AR splice variants lacking the androgen ligand-binding domain contribute to NHT resistance (5, 6). Accumulating evidence suggests that Abiraterone and Enzalutamide are not effective in AR-V7-positive patients (7–9). However, AR-V7-positive patients showed a better response to taxane chemotherapy (8, 10, 11). Therefore, AR-V7 may guide treatment decisions for CRPC patients (12).

Several studies have reported that AR-V7 is unable to predict the effectiveness of NHT in CRPC (13, 14). Moreover, AR-V7 positivity has been associated with unfavorable baseline characteristics and a higher tumor burden (10, 15). AR-V7 mediates taxane resistance *via* several mechanisms. Previously, high expression of AR-V7 was linked to NHT resistance, but its expression level declined after chemotherapy (7, 8, 16–21). In recent years, the prognostic value and clinical utility of AR-V7 in NHT and chemotherapy are deeply investigated. Promising data support AR-V7 as a treatment selection marker in therapeutic strategies for mCRPC patients. However, due to the diversities of patient cohorts and sample characteristics, the varieties of detection methods and different sample types (**Tables 1**, **2**), and the various definitions of AR-V7 positivity and clinical endpoints (**Supplementary Tables S1, S2**), the clinical utility of AR-V7 as a treatment selection marker in CRPC still needs to be further evaluated.

**Table 1:** Characteristics of studies included in the change of AR-V7-positive proportion after treatment meta-analysis.

Author	Year	Country	Study design	Characteristics						
				AR-V7 detection assay	Therapy strategy	Patients (n)	Age (range)	Gleason score(range)	Median PSA(ng/ml) at sampling(range)	Follow-up time(month) Median(range)
Antonarakis et al. (9)	2017	USA	prospective	CTC mRNA	ABT or ENZ	53 CTC–	70	≥8 (68.0%)	13.7	28.7
						113 CTC+/AR-V7–	71	≥8 (60.0%)	31.4	29.5
						36 CTC+/AR-V7+	70	≥8 (83.0%)	92.0	11.2
Nakazawa et al. (18)	2015	USA	prospective	CTC mRNA	First-line ADT/ABT/ENZ/docetaxel/cabazitaxel	14	65 (50–82)	≤7 (92.9%) ≥8 (0%) Unknown (7.1%)	58.7 (2.2–895)	11 (6–18)
Sieuwerts et al. (21)	2019	Netherlands	prospective	CTC mRNA	cabazitaxel	52	69(SD=7)		209 IQR(72–510)	
Zadra et al. (22)	2019	USA	retrospective	Immuno- fluorescence	ABT or ENZ	55	55			
Armstrong et al. (17)	2019	USA	prospective	CTC mRNA	ABT or ENZ	118	73(45-92)	≥8 (58%)	19 (0.08-4194)	19.6
Antonarakis et al. (7)	2014	USA	prospective	CTC mRNA	ABT	31	69(48–79)	≤7 (26.7%) ≥8 (73.3%)	37.8 (2.2–2045.0)	4.6 (0.9–8.2)
					ENZ	31	70(56–84)	≤7 (40%) ≥8 (60%)	44.3 (4.3–3204.2)	5.4 (1.4–9.9)
Antonarakis et al. (8)	2015	USA	prospective	CTC mRNA	Docetaxel or cabazitaxel	37	67 (46–82)	≤7 (17%) ≥8 (83%)	126 (0.1–2270)	7.7 (0.7–19.0)
Welti et al. (20)	2016	UK	retrospective	IHC	ABT/ENZ	35	67.5 (IQR64.2-75.3)			
Sharp et al. (23)	2019	USA	prospective	IHC	ABT/ENZ	160	68.5 IQR(63.9-73.1)		230.5 IQR(77.0-591.5)	

PCa, prostate cancer; CRPC, castration resistance prostate cancer; PSA, prostate specific antigen; IQR, inter quartile range; ABT, Abiraterone; ENZ, Enzalutamide; ADT, androgen deprive therapy; IHC, Immunohistochemistry; CTC, circulating tumor cell.

**Table 2:** Characteristics of studies and patients included in the PSA response, PFS and OS meta-analysis.

Study	Year	Country	Study design	AR-V7 detection assay	Patients characteristics							
					Treatment	Patients (n)	Age(range)	Gleason score (%)	Tumor stage at diagnosis (%)	Baseline PSA (ng/ml) median (range)	Baseline alkaline phosphatase (U/L)	Follow-up time(month)Median(range)
Antonarakis et al. (7)	2014	USA	prospective	CTC mRNA	Abiraterone	31	69 (48–79)	≤7 (26.7%) ≥8 (73.3%)	T1/T2 (26.7%) T3/T4 (61.3%)	37.8 (2.2–2045.0)	118 (59–1348)	4.6 (0.9–8.2)
					Enzalutamide	31	70 (56–84)	≤7 (40%) ≥8 (60%)	T1/T2 (54.8%) T3/T4 (45.2%)	44.3 (4.3–3204.2)	108 (58–872)	5.4 (1.4–9.9)
Antonarakis et al. (8)	2015	USA	prospective	CTC mRNA	Docetaxel or cabazitaxel	37	67 (46–82)	≤7 (17%) ≥8 (83%)	T1/T2 (38.0%) T3/T4 (62.0%)	126 (0.1–2270)	161 (53–1243)	7.7 (0.7–19.0)
Scher et al. (24)	2016	USA	cohort study	CTC Immunofluorescent Staining	NHT	130	68.5 (45–87)	–	–	28.0 (0.1–2454.5)	208 (123–1293)	–
					Taxane Therapy	63	68 (48–91)	–	–	99.5 (0.1–3728.2)	251.5 (141–1004)	–
Steinestel et al. (25)	2015	Germany	prospective	CTC mRNA	Enzalutamide or Abiraterone	24	75 (53–87)	≤7 (41.3%) ≥8 (58.7%)	–	96.5 (0.1–4282)	–	–
Todenhofer et al. (26)	2016	Canada	prospective	CTC mRNA	Abiraterone	37	70 (53–87)	–	–	–	116 (45–1869)	–
Welti et al. (20)	2016	UK	retrospective	IHC	NHT or chemotherapy	35	67.5 (IQR64.2-75.3)	–	–	–	142.0 (69.5–448.5)	–
Onstenk et al. (13)	2015	Netherlands	prospective	CTC mRNA	Cabazitaxel	29	70 (SD ± 7)	–	–	321 (IQR76-649)	163 (106-375)	7 (2-27)
Qu F et al. (27)	2016	USA	retrospective	CTC mRNA	Abiraterone	81	68.3 (46-89)	≤7 (41.9%) ≥8 (49.4%) Unknown (8.6%)	–	16.4 (0.1-972.1)	–	29.7 (3.6-47.5)
					Enzalutamide	51	69.0 (5-88)	≤7 (47.1%) ≥8 (41.2%) Unknown (11.8%)	–	45.5 (0.3-1148.4)	–	23.9 (0.9-48.3)
Antonaraki et al. (9)	2017	USA	prospective	CTC mRNA	Enzalutamide or Abiraterone	53 CTC–	70	≥8 (68.0%)	–	13.7	80	28.7
						113 CTC+/AR-V7–	71	≥8 (60.0%)	–	31.4	96	29.5
						36 CTC+/AR-V7+	70	≥8 (83.0%)	–	92.0	120	11.2
Nakazawa et al. (18)	2015	USA	prospective	CTC mRNA	NHT or chemotherapy	14	65 (50–82)	≤7 (92.9%) ≥8 (0%) Unknown (7.1%)		58.7 (2.2–895)	127 (52–838)	11 (6–18)
Del Re et al. (28)	2017	Italy	prospective	Plasma exosomal RNA	Enzalutamide or Abiraterone	36	66 (51-81)	≤7 (44%) ≥8 (53%) Unknown (3%)	T1/T2 (8.0%) T3/T4 (36.0%)	26.3 (0.63–4581)	180 (49–917)	9 (2.0-31.0)
Zhu et al. (24)	2018	USA	retrospective	RISH	Enzalutamide or Abiraterone	28	64 (52-86)	≤7 (21.4%) ≥8 (78.6%)		59.6 (0.7-6746.8)		
		UK	retrospective	RISH	Enzalutamide or Abiraterone	16	72.3(48.8-79.4)	≤7 (25%) ≥8 (50%) Unknown (25%)		177.0 (2.6-4098.0)		
To et al. (14)	2018	Australia	prospective	whole blood mRNA	Enzalutamide or Abiraterone	9 AR-V positive	77 (46–89)	≤7 (44.4%) ≥8 (55.6%)		49.2 (3–703)		
						28 AR-V negative	75.5 (52–89)	≤7 (14.3%) ≥8 (46.4%) Unknown (39.3%)		42.5 (0.8–588)		
Seitz et al. (29)	2017	Germany	prospective	whole blood mRNA	Enzalutamide or Abiraterone	85	71 (66–74)			211 (IQR29–768)		7.6 (IQR4.7–12.7)
De Laere et al. (30)	2017	Belgium	retrospective	CTC mRNA	Enzalutamide or Abiraterone	17						
Scher et al. (31)	2017	USA	prospective	CTC mRNA	Enzalutamide or Abiraterone	161	68 (45–91)	8 (range 5–10)		37.7 (0.1–3728.2)	111 (25–2170)	11 (1–30)
Takeuchi et al. (13)	2016	Japan	cohort study	whole blood mRNA	Enzalutamide or Abiraterone	43	73 (59–88)	≤7 (20.9%) ≥8 (72.1%) Unknown (7.0%)		130 (5.3–9529)		
Okegawa et al. (32)	2018	Japan	retrospective	CTC mRNA	Enzalutamide or Abiraterone	49 CTC−	69	≥8 (81.6%)		75.7	317	20.7 (3.0-37.0)
						23 CTC+ AR-V7−	71	≥8 (91.3%)		71.5	323	
						26 CTC+ AR-V7+	72	≥8 (96.2%)		79.1	378	
Kohli et al. (33)	2017	USA	prospective	CTC mRNA and biopsies mRNA	Abiraterone	78						
Tommasi et al. (34)	2018	Italy	prospective	CTC mRNA	Enzalutamide or Abiraterone, LHRH, Chemotherapy	44	71.5 (55-87)	≤7 (6.8%) ≥8 (81.8%) Unknown (11.4%)	T1/T2 (15.9%) T3/T4 (79.5%) Unknown (4.5%)	39 (0.005-2896)	–	20.5
Armstrong et al. (17)	2019	USA	prospective	CTC mRNA	Enzalutamide or Abiraterone	118	73 (45-92)	≥8 (58%)	M1(32%)	19 (0.08-4194)	40% Elevated	19.6
Sharp et al. (19)	2019	USA	prospective	IHC	Enzalutamide or Abiraterone, Chemotherapy	160	68.5 IQR(63.9-73.1)		M1b(67%) M1c(21%)	230.5 IQR(77.0-591.5)	127.0 IQR(72.3-332.5)	
Sieuwerts et al. (21)	2019	Netherlands	prospective	CTC mRNA	cabazitaxel	52	69(SD=7)			209 IQR(72–510)	174 IQR(98–339)	
Cattrini et al. (35)	2019	Italy	prospective	CTC mRNA	Enzalutamide,Abiraterone or Docetaxel	39	72 (56-84)		M1b(79.5%) M1c(17.9%)	35.2 (0.33–4688)		
Chung et al. (36)	2019	USA	prospective	CTC mRNA	Enzalutamide or Abiraterone	37	72 (67-79)	≤7 (43.2%) 8(8.1%) ≥9 (46%) Unknown (2.7%)	N1(64.9%) M1b(89.2%) M1c(27%)	20.9 IQR(11.6-96.8)	102.0 IQR(80.5-170.5)	11.43 (IQR: 4.73-21.3)
Del Re et al. (37)	2019	Italy	retrospective	Plasma exosomal RNA	First time Enzalutamide or Abiraterone	46		≤7 (43.5%) ≥8 (47.8%) Unknown (8.7%)	T1/T2 (13%) T3/T4 (13%) N1(21.8%) M1(21.8%)			
					second time Enzalutamide or Abiraterone	27		≤7 (52%) ≥8 (38%) Unknown (10%)	T1/T2 (11.1%) T3/T4 (25.9%) N1(14.8%) M1(40.8%)			
El-Heliebi et al. (38)	2018	Austria Germany Netherlands Sweden	prospective	CTC FISH	Enzalutamide, Abiraterone or Taxane	31	70.5 (42-83)	≤7 (38.7%) ≥8 (51.6%)	T1/T2 (22.6%) T3/T4 (45.2%)	48.81 (0.8-4623)		
Tagawa et al. (15)	2019	USA	prospective	CTC mRNA	Docetaxel or Cabazitaxel	54	71 (53–84)	≤6 (13.7%) 7(25.5%) ≥8 (60.8%)	N1 (51.9%) M1b(90.7%) M1c(40.7%)	92.1 (2.4–1558)	217.8 (SD=260.35)	
Worroll et al. (39)	2019	USA	prospective	CTC Immunofluorescence	Taxane	15						
Belderbos et al. (40)	2019	Netherlands	prospective	CTC mRNA	Enzalutamide, Abiraterone or Cabazitaxel	94	69 IQR (65-75)			186 IQR (67-356)		
Sharp et al. (23)	2019	UK/USA	prospective	CTC mRNA /IHC	Enzalutamide, Abiraterone or Taxane	95 CTC-	71.0 IQR (66.8– 75.6)		M1b(74.7%) M1c(17.9%)	110.0 IQR (29–300.5)	83.0 IQR (66.0–163.0)	
						86 CTC+ ARV7-	69.6 IQR (64.9– 72.3)		M1b(86.1%) M1c(24.4%)	147.0 IQR (51.0–345)	111.5 IQR (76.3–200.5)	
						96 CTC+ ARV+	70.4 (65.3– 74.6)		M1b(84.4%) M1c(24.0%)	244.5 IQR (109.3–746.8)	180.0 IQR (93.8–346.0)	
Maillet et al. (41)	2019	France	prospective	CTC mRNA	Enzalutamide or Abiraterone	41	73	≥8 (56%)	M1(29%)	35		10.5(95%CI 8.7-13.7)
Graf et al. (42)	2019	USA	prospective	CTC mRNA	Enzalutamide/Abiraterone or Taxane	193	69 IQR (62.5– 75)		M1b(89%) M1c(22%)	50.4 IQR (18.2–211.2)	111 IQR (80–199.5)	
Erb et al. (43)	2020	Germany	prospective	CTC IHC	Enzalutamide/Abiraterone or Taxane	26	74.3 ± 9		N1(65.4%) M1b(84.6%) M1c(19.2%)			
Kwan et al. (44)	2020	Australia	prospective	whole blood mRNA	Enzalutamide/Abiraterone or Taxane	115	*72 (46*–*91)*	≤7 (24%) ≥8 (50%)	M1(53%)	*42 (0.51*–*2719)*	*131 (45*–*5918)*	*15.5* *(1.4*–*29)*

IQR, inter quartile range; SD, standard deviation; AR-V7, androgen receptor splice variant 7; CTC, circulating tumor cell; PSA, prostate specific antigen; ADT, androgen deprivation therapy; NHT, novel hormonal therapy; LHRH, luteinizing hormone releasing hormone.

In this review, a meta-analysis was conducted on 36 studies to explore the prognostic value of AR-V7 in CRPC patients treated with NHT or chemotherapy by the PSA response, OS and PFS. In addition, the conversion of AR-V7 positive proportion after NHT or chemotherapy was further analyzed.

## Methods

### Retrieval Strategy

This meta-analysis was conducted based on the PRISMA statement and registered at PROSPERO with registration number CRD42020161618 (45). A literature search was performed on PubMed, the Web of Science, and Embase from inception to February 2020. The main search strategy was as follows: (prostate cancer OR prostate tumor OR prostate neoplasm OR prostate carcinoma) AND (AR-V7 OR AR3 OR androgen receptor splicing variant 7 OR androgen receptor 3). Bibliographic references of selected articles were examined to identify additional articles that met the selection criteria. Two reviewers independently verified the studies for eligibility and any discrepancies were resolved by a third reviewer.

### Selection Criteria

Retrieved titles and abstracts were screened to eliminate duplicates, and full texts in the selected articles were reviewed and matched against the inclusion criteria. Articles eligible for inclusion met the following criteria: 1) The study reported on CRPC and AR-V7; 2) The results were expressed as a positive rate in percent of AR-V7 before and after treatment in CRPC. Other included results were the PSA response rate, PFS, or OS after NHT or chemotherapy; 3) The results were reported from clinical trials including RCTs and nonrandomized studies. Studies were excluded if: 1) treatment was neither novel hormonal therapy nor chemotherapy, or not clearly mentioned; 2) studies reported only the AR-V7-positive proportion before or after treatment in CRPC patients; 3) studies lacked results of therapy response rate, PFS, or OS; 4) studies involved non-human subjects; 5) studies were published in languages other than English except when a translation was provided; 6) case reports, comments, editorials, letters, or reviews. Notably, when more than one publication was retrieved from the same trial, the most recent or most complete report was used.

### Data Collection and Study Quality

1) The following baseline patient characteristics were collected for each eligible trial: age, tumor stage, Gleason score, baseline PSA and alkaline phosphatase, and the median time from diagnosis to sampling. 2) The description of the study design included the author’s name, year, country, study design, type of therapy, description of interventions, and outcome measures. 3) The number of patients assigned to different treatment regimens and follow-up duration. 4) The AR-V7-positive percentage before and after treatment in CRPC samples and the methods of detection. 5) The number and proportion of patients with therapy response to NHT or chemotherapy and its detailed definition. 6) The prognosis outcome data were the hazard ratios (HRs) with 95% confidence intervals (CIs) and P-values of PFS, detailed in the subgroup analysis of NHT or chemotherapy. 7) For OS, HRs with 95% CIs and P-value, detailed in the subgroup analysis of NHT or chemotherapy.

### Statistical Methods

Summary statistics were generated using Review Manager Software (RevMan v.5.3; The Nordic Cochrane Center, Copenhagen, Denmark). All patients were included and analyzed on an intention-to-treat basis. The AR-V7-positivity conversion after treatment and the association between AR-V7 expression and the response rate of NHT or chemotherapy were assessed. The primary endpoints of the meta-analysis were OS and PFS. Patients were grouped by AR-V7 status and compared in terms of the PSA response rates, PFS, and OS after NHT or chemotherapy in CRPC. The summary measure for OS and PFS was HR (95% CI). Statistical differences among studies were evaluated using the chi-square test and the I^2^ statistic. Odds ratio (OR) and hazard ratio (HR) estimates were weighted by the Mantel-Haenszel method. The pooled effect was calculated using either the fixed effects model (I^2^ < 50%) or the random-effects model (I^2^ ≥ 50%) based on heterogeneity level. All statistical tests were bilateral with a significance *p* value of 5%.

## Results

### Study Characteristics and Quality

**Figure 1:**
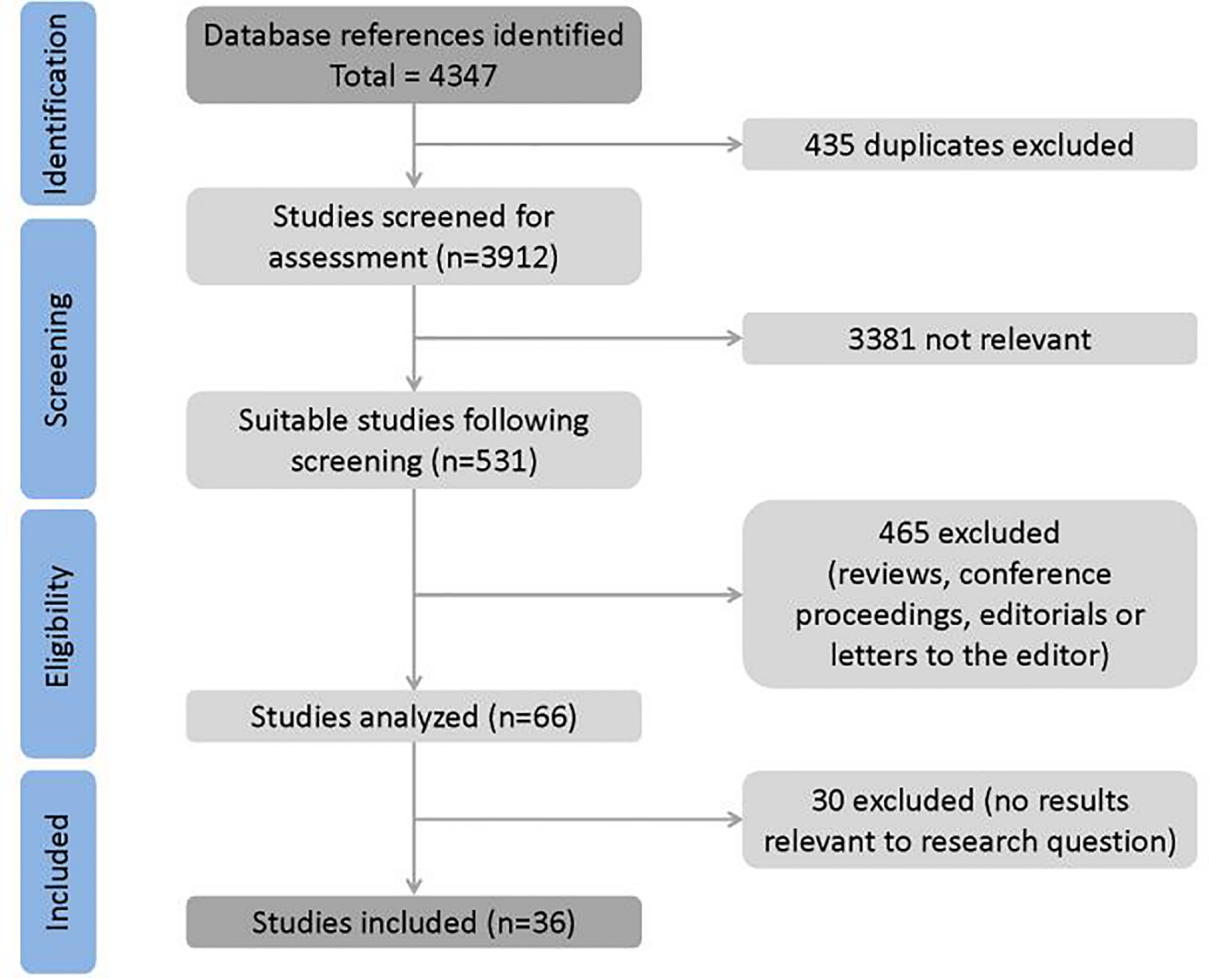
Study selection process.

Article selection process is shown in **Figure 1**. A total of 4347 full-text papers were identified from which 4313 were excluded. Notably, 435 studies were duplicate, 3,381 were not relevant to the research question, 465 were conference abstracts, reviews, letters, and editorials whose quality could not be assessed, and 30 studies did not contain relevant results. No study was identified from reference lists. Eventually, 36 trials were included in the meta-analysis.

### Patient Characteristics

Nine trials including 897 patients were used to compare AR-V7 conversion after treatment. Their clinicopathological features are listed in **Table 1**. The sample type and AR-V7 detection methods are detailed in **Supplementary Table S1**. A total of 1,643 patients from 24 studies were enrolled for therapy response comparison; 17 studies including 1,499 patients were used for the comparison of PFS; 1,726 patients from 17 studies were enrolled for OS comparison. Detailed features of these studies are highlighted in **Table 2**. The definitions of PSA response, PFS, and OS were inconsistent across studies and was displayed in **Supplementary Table S2**.

### AR-V7 Positivity Conversion After Treatment

**Figure 2:**
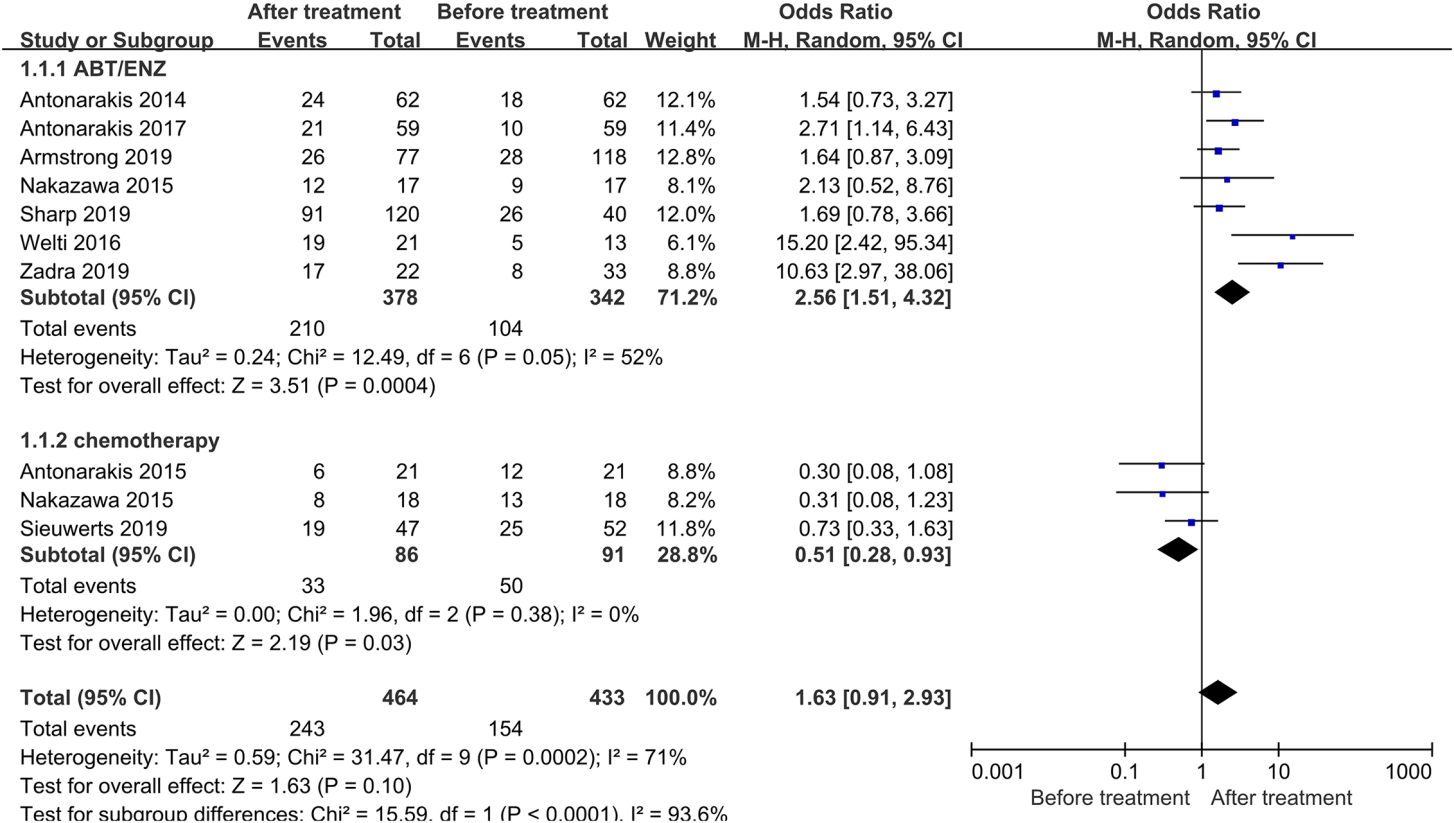
Forest plots of AR-V7-positive proportion conversion after treatment from nine studies. AR-V7-positive proportion before and after treatment were calculated using random-effect models. The bars indicate 95% CIs. AR-V7, androgen receptor splicing variant 7; ABT, Abiraterone; ENZ, Enzalutamide; CI, confidence interval; OR, odds ratio.

A total of 897 patients from nine trials were enrolled. Of note, 154 out of 433 patients were AR-V7 positive before treatment, however, the proportion converted to 243 out of 464 after treatment. Subgroup analysis of different treatment strategies was performed (**Figure 2**). A significant study heterogeneity was detected (I^2^ = 71%, P<0.001), and thus a random-effects model was used. The proportion of AR-V7 positivity significantly increased after Abiraterone or Enzalutamide treatment (OR 2.56, 95% CI 1.51–4.32, P<0.001) whereas, it significantly declined after chemotherapy (OR 0.51, 95% CI 0.28–0.93, P=0.003).

### The Relationship Between AR-V7 Expression and PSA Response in CRPC Patients

**Figure 3:**
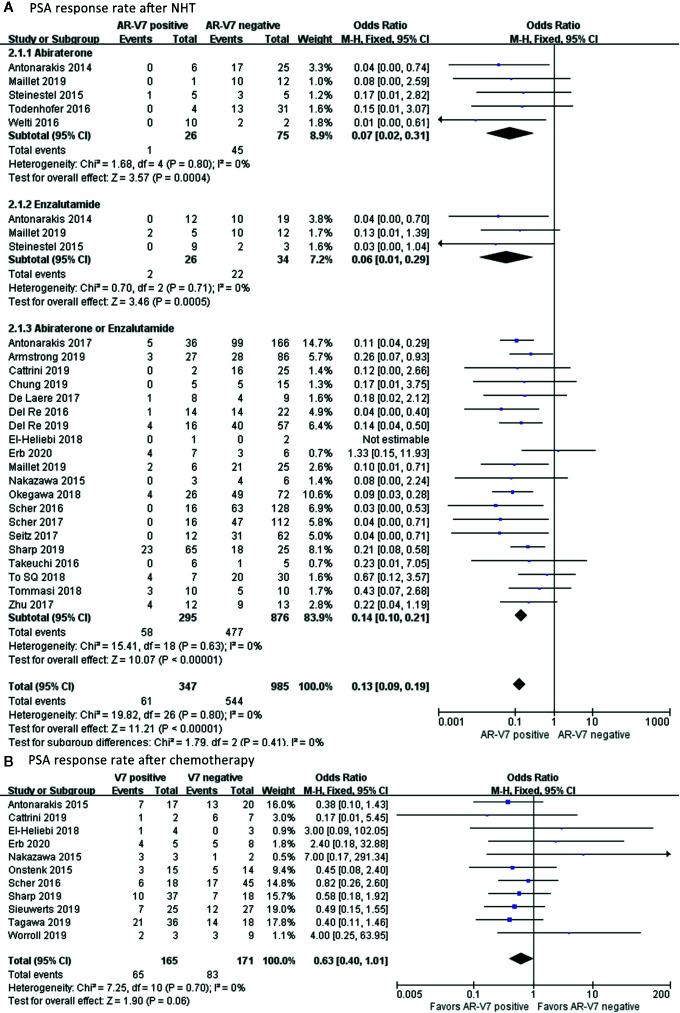
Forest plots of PSA response rate after NHT and chemotherapy. The PSA response rate in AR-V7-positive and AR-V7-negative patients after NHT and chemotherapy was calculated using fixed-effect models. **(A)** PSA response rate after NHT. **(B)** PSA response rate after chemotherapy. The bars indicate 95% CIs. NHT, novel hormonal therapy; ABT, Abiraterone; ENZ, Enzalutamide; PSA, prostate specific antigen; AR-V7, androgen receptor splicing variant 7; CI, confidence interval.

In total, 61 out of 347 AR-V7-positive men showed good response to NHT whereas, 544 out of 985 AR-V7-negative CRPC showed good response to PSA (**Figure 3A**). AR-V7-positive patients had significantly lower PSA response after NHT treatment compared to AR-V7-negative (OR 0.13, 95% CI 0.09–0.13, P<0.001). However, no significant heterogeneity was observed among the studies in this effect (I^2^ = 0.0%, P=0.80). In subgroup analysis, the OR of a PSA response in AR-V7-positive CRPC was 0.07 (95% CI 0.02–0.31, P<0.001) for Abiraterone-treated group, 0.06 (95% CI 0.01–0.29, P<0.001) for Enzalutamide group, and 0.14 (95% CI 0.10–0.21, P<0.001) for Abiraterone or Enzalutamide subgroup. Similarly, 65 out of 165 AR-V7 positive patients showed therapeutic response to chemotherapy whereas, in AR-V7 negative patients, the proportion was 48.5% (83 of 171), (**Figure 3B**). Moreover, the PSA response rate was decreased in AR-V7 positive patients (OR 0.63, 95% CI 0.40–1.01, P=0.06). A fixed-effects model was applied since there was no discrepancy between studies (I^2^ = 0.0%, P=0.70).

### The Effect of AR-V7 Expression on Progression-Free Survival in CRPC Patients

**Figure 4:**
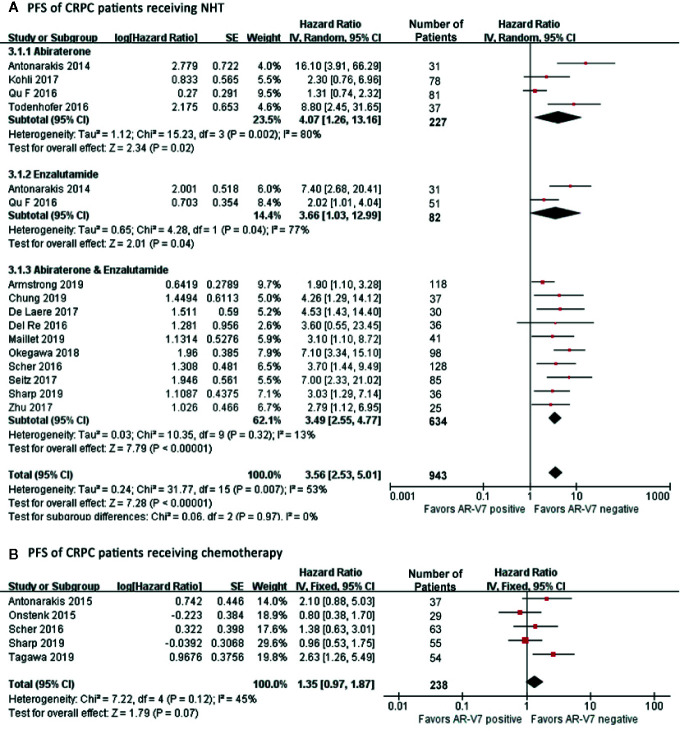
Forest plots of hazard ratios (HRs) for PFS of NHT and chemotherapy in CRPC patients. Pooled HRs were calculated using random effect for NHT and fixed effect model for chemotherapy. The bars indicate 95% CIs. **(A)** PFS of CRPC patients receiving NHT. **(B)** PFS of CRPC patients receiving chemotherapy. NHT, novel hormonal therapy; AR-V7, androgen receptor splicing variant 7; CI, confidence interval; PFS, progression free survival.

AR-V7 negative patients benefited more from NHT in terms of PFS compared with the positive patients (HR 3.56, 95% CI 2.53–5.01, P<0.001), (**Figure 4A**). Notable study discrepancy was tested (I^2^ = 53%, P=0.007) where a random effect model was applied for this analysis. Further subgroup analysis revealed that AR-V7 positive patients had worse PFS following Abiraterone treatment (HR 4.07, 95% CI 1.26–13.16, P=0.02), Enzalutamide treatment (HR 3.66, 95% CI 1.03–12.99, P=0.04), and Abiraterone/Enzalutamide treatment (HR 3.49, 95% CI 2.55–4.77, P<0.001). However, patient with AR-V7-negative status tended to have a better PFS although not significantly (HR 1.35, 95% CI 0.97–1.87, P=0.07), (**Figure 4B**). There was no heterogeneity between the studies (I^2^ = 45%, P=0.12), therefore, the fixed-effects model was applied.

### The Effect of AR-V7 Expression on Overall Survival of CRPC Patients

**Figure 5:**
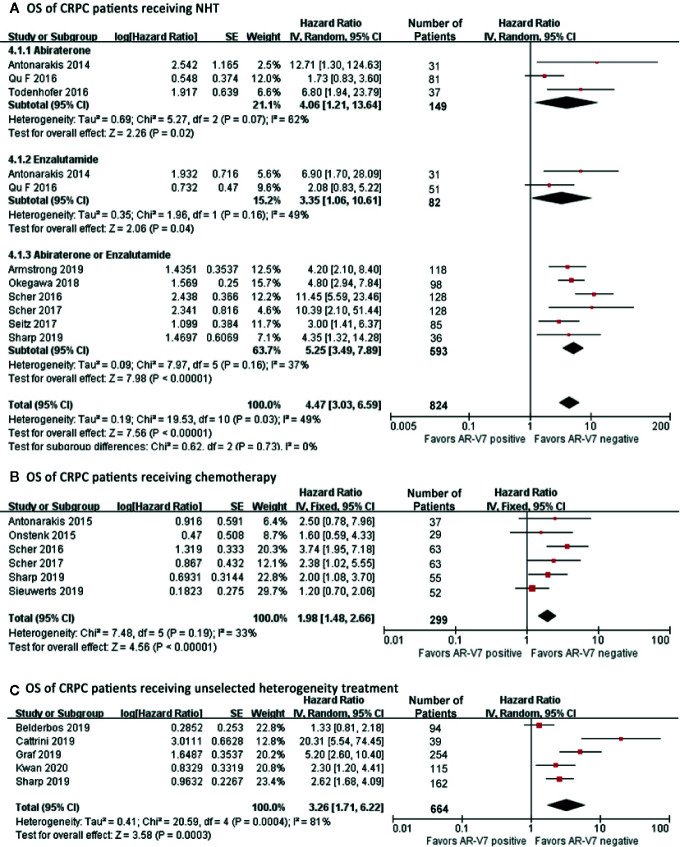
Forest plots of hazard ratios for OS in CRPC patients received NHT, chemotherapy or non-standardized treatment. Pooled HRs were calculated using random effect model. The bars indicate 95% CIs. **(A)** OS of CRPC patients receiving NHT. **(B)** OS of CRPC patients receiving chemotherapy. **(C)** OS of CRPC patients receiving non-standardized treatment. AR-V7, androgen receptor splicing variant 7; CI, confidence interval; OS, overall survival.

Among all patients who received NHT, AR-V7 negative patients had the best OS (HR 4.47, 95% CI 3.03–6.59, P<0.001), (**Figure 5A**). A significant study difference was detected (I^2^ = 49%, p=0.03), thus a random effect model was applied. Further subgroup analyses indicated that AR-V7 positive patients had a worse OS after Abiraterone treatment (HR 4.06, 95% CI 1.21-13.64, P=0.02), Enzalutamide (HR 3.35, 95% CI 1.06–10.61, P=0.04), and Abiraterone/Enzalutamide treatment (HR 5.25, 95% CI 3.49–7.89, P<0.001). For patients who received chemotherapy, AR-V7 positive patients had a worse OS than negative patients (**Figure 5B**, HR 1.98, 95% CI 1.48–2.66, P<0.001). A similar result was observed for non-standardized treatment (**Figure 5C**, HR 3.26, 95% CI 1.71–6.22, P<0.001).

## Discussion

This meta-analysis aimed to explore the prognosis value of AR-V7 in CRPC patients treated with NHT or chemotherapy. Several reports have indicated that AR-V7 can potentially induce and promote NHT resistance in CRPC and predict the prognosis of CRPC (6, 46, 47). It has been shown that taxane can maintain tubulin stability, inhibit cell mitosis and thereby induce apoptosis by promoting tubulin polymerization, decreasing AR nuclear localization, and inhibit depolymerization (15). Androgen receptor variants contain DNA binding domain and the hinge region. They can decrease taxane sensitivity by altering the inhibit ability of taxane of AR nuclear localization. Unlike ARv567, ARV7 lacks the hinge region, thus cannot co-sediment with microtubules or coprecipitate with dynein motor protein, it is therefore unaffected by taxane treatment (48). Although AR-V7 contributes to NHT resistance and chemotherapy, its predictive role in CRPC treatment has not been validated.

Our results show that the conversion of AR-V7 positivity increased in CRPC patients after NHT but decreased after chemotherapy. The change in AR-V7 expression was first reported by Antonarakis et al. in NHT-treated CRPC patients detected by circulating tumor cell (CTC) mRNA (7). Clinical reports have indicated that the AR-V7 positivity is elevated after NHT (8, 9, 17, 18, 20). A similar observation has been reported after taxane treatment, but not as statistically significant as NHT (8, 18, 21). The present study shows that taxane chemotherapy decreased AR-V7 expression.

The different conversion of AR-V7 expression after NHT and chemotherapy indicates different treatment mechanisms. These findings show that taxane-based therapeutic combination may be effective against drug resistance in CRPC (49), particularly for AR-V7 positive patients. However, due to the various chemotherapeutic agents and the different AR-V7 detecting assays among studies, these results need to be verified. Therefore, further research is needed to elucidate the conversion mechanism of AR-V7 expression during treatment.

Further analysis showed that, among patients who received NHT, the PSA-response proportion was lower in AR-V7-positive patients than negative. This indicated that AR-V7 could potentially predict NHT resistance. In our previous study, taxane-based chemotherapy tended to be highly beneficial than NHT in AR-V7 positive patients whereas the efficacy of NHT and taxane in AR-V7 negative patients was similar (12). Recent trials reported that AR-V7-positive CRPC patients show a lower PSA response to chemotherapy than AR-V7-negative patients (15, 21, 23, 35) whereas others have reported opposite results (38, 39). A previous study demonstrated that AR-V7 derived from CTC was not associated with primary resistance to taxane-based chemotherapy in patients with metastatic CRPC (8). By contrast, our results indicate a significantly lower PSA response to chemotherapy in AR-V7-positive patients. The prior-treatment of enrolled patients was diversiform, and the effect of prior NHT on the following chemotherapy was unclear. Further research is required to elucidate the relationship between AR-V7 and taxane chemotherapy resistance.

Sub-analysis was carried out to delineate the association of AR-V7 expression with the PFS and OS. The NHT group was further sub-classified into Abiraterone, Enzalutamide, or a combination of both. Of all patients receiving NHT, AR-V7-positive patients had worse PFS and OS than AR-V7-negative patients. Since the first report by Antonarakis et al. that AR-V7 in CTCs was associated with NHT resistance and poor survival in CRPC patients (7, 8, 11), the prognostic value of AR-V7 of CRPC patients receiving NHT has been extensively studied. Additionally, a large prospective study by Antonarakis et al. examined the negative prognostic effect of CTC-based AR-V7 testing in patients with CRPC receiving Abiraterone or Enzalutamide (9). Although AR-V7 showed its potential clinical utility as a prognostic biomarker in CRPC, the diverse findings on the predictive value and specificity of different AR-V7 detection methods limited its clinical validation. Discordant CTC AR-V7 results and AR-V7 protein expression in matched, same-patient biopsies were reported by Sharp et al. whereby AR-V7 positivity was associated with higher CTC counts which may have confounded outcome analyses (23). The prognostic role of AR-V7 in CRPC patients receiving chemotherapy has been a subject of research. Previous trials indicated that AR-V7 in CTCs from mCRPC was not associated with primary resistance to taxane chemotherapy and potentially responded better to taxane than NHT (8, 50). By contrast, some studies reported that the AR-V7 did not affect the outcome of taxane treatment in patients with mCRPC (15, 21). In this study, a worse outcome was observed in AR-V7 positive patients receiving chemotherapy with a nearly statistically significant PFS (HR 1.35, 95% CI 0.97–1.87, P=0.07) and remarkably shorter OS (HR 1.98, 95% CI 1.48–2.66, P<0.001). Given the inconsistent results from previous studies, whether AR-V7 could be applied to guide treatment decisions in CRPC is unclear. A report by Graf et al. indicated that doctors prefer taxane to NHT for patients with a highly aggressive disease or who have received NHT previously, which might result in treatment selection tendency. After adjusting physician propensity, no marked differences in OS were observed between taxane- and NHT-treated patients (42). Elsewhere, nuclear CTC expression of AR-V7 protein was associated with better survival in mCRPC patients receiving taxane therapy than those receiving NHT (11). Therefore, multiple therapeutic modalities may be applied simultaneously to effectively reverse resistance in AR-V7 positive CRPC (19). Moreover, AR-V7 should be consistently monitored during treatment and more effective target agents for AR-V7 are needed. Further prospective validation studies are required to determine the prognostic role of AR-V7 in CRPC (51).

This study is limited by the different comprised sample sizes ranging from 14 to 277 patients, and the statistical power is restricted by the following: First, small sample size and limited follow-up time of some studies would yield less reliable results due to the size effect. The risk of publication bias among the studies cannot be ignored. We plotted low statistical power trials in **Supplementary Figures S1–S5** by asymmetrical distribution of funnel plots for further investigation. Second, research designs of the included studies are not unified. Most trials are single-centered and thus selection bias cannot be ruled out. Patient selection criteria are highly variable among different studies. Third, different assays were used to measure AR-V7 expression ranging from qRT-PCR, immunohistochemistry (IHC), fluorescence in-situ hybridization (FISH) and RNA in-situ hybridization (RISH). These detection methods carry different advantages and disadvantages as described in previous reviews (51, 52). Therefore, positivity rate may vary across studies because of different cutoff values. The sensitivity and specificity of tissue-based detection are not optimal because of the nonspecific detection of nuclear AR-V7 pre-mRNA by RISH and the nonspecific binding reaction of AR-V7 anti-body. Moreover, although it is feasible to measure AR-V7 and other AR aberrations using blood-based assays, it is still necessary to warrant the clinical validation of individual and integrated assays in the future (53). For CTC mRNA detection, CTC AR-V7 status was discordant between CellSearch and AdnaTest at low AR-V7 mRNA expression level which may confound outcome analyses (23). The sample type and AR-V7 test methods applied in the enrolled studies were detailed in **Supplementary Table S1**. Fourth, patients’ characteristics such as tumor stage, metastasis and endpoint definitions are different. This may result in a possible discrepancy among studies (**Supplementary Table S2**). Last but not the least, physicians tended to choose chemotherapy rather than NHT for patients with more advanced disease or who had already received NHT as immediate prior therapy, and AR-V7 positivity was associated with highly aggressive prostate cancer and tumor burden: this may confound the results and generate bias. Therefore, AR-V7 was not extensively and sufficiently studied in prostate cancer.

We attempted to minimize these limitations. First, we performed a comprehensive systematic literature search on major online databases with a reproducible strategy to minimize publication bias. Second, although selection bias could not be eliminated, strict inclusion criteria were applied to minimize bias caused by different AR-V7 test assays, treatment response definition, and therapy history. Third, a subgroup analysis was performed according to different therapies and it was reported that the correlation between AR-V7 and therapy outcomes was consistent across the subgroup stratified by NHT and chemotherapy. Fourth, the type of trial, tumor stage, AR-V7 test assay, therapy strategy, prior-treatment PSA, and surveillance time are detailed in tables for further analysis and reference. Biomarkers that provide accurate prognostic information for CRPC are urgently required. Several randomized clinical trials (RCTs) have indicated the benefits of early inclusion of docetaxel to androgen deprivation therapy in hormone-sensitive metastatic prostate cancer (54–57). Besides, in mCRPC, clinical data favored taxane over NHT in the second-line treatment setting for the optimal patient benefit (58). Recent research reported that novel taxane-based combination therapies potentially improve outcomes (49). However, validated biomarkers are desired for selecting suitable patients due to the risk of enhanced toxicity. Various prognostic markers have been identified for CRPC (59) but none can be applied in selecting a treatment strategy. This meta-analysis suggested that AR-V7 may be a potential therapeutic target and prognostic biomarker in CRPC patients. There is an urgent need for more prospective trials to verify the utility of AR-V7 as a biomarker in CRPC. Additionally, researches on biomarker-driven or biomarker-stratified clinical trials are required to improve AR-V7 evaluation following the findings from this meta-analysis.

## Conclusion

The significant prognostic value of AR-V7 in CRPC patients treated with NHT or chemotherapy was determined in this meta-analysis. The AR-V7 expression significantly increased after NHT but remarkably decreased after chemotherapy. Moreover, AR-V7 positive CRPC patients portend worse prognosis of NHT with a lower therapy response, poorer PFS, and OS. Besides, for chemotherapy, AR-V7 positive patients acquired fewer benefits of prognosis with a lower PSA response, shorter OS, and a nearly significantly worse PFS. Based on these results, AR-V7-positive CRPC should be considered as a specific subtype with a worse prognosis and be treated with a highly active anti-tumor therapy. Furthermore, the AR-V7 status should be consistently monitored and AR-V7 targeted therapy strategies are needed. However, cross-institutional studies are still needed to validate AR-V7 as a selection treatment marker. Future studies should aim to improve AR-V7 detection to validate the clinical utility in CRPC.

## Data Availability Statement

All datasets presented in this study are included in the article/**Supplementary Material**.

## Author Contributions

ZW: project development, data collection, data analysis, manuscript writing. HS: data collection, data analysis, manuscript writing. NM: data collection, data analysis. QL: data collection, data analysis. YM: data collection, data analysis. CW: project development, manuscript editing. LX: project development, manuscript editing. All authors contributed to the article and approved the submitted version.

## Conflict of Interest

The authors declare that the research was conducted in the absence of any commercial or financial relationships that could be construed as a potential conflict of interest.
