# Supplementary material for: The Prognostic Value of Androgen Receptor Splice Variant 7 in Castration-Resistant Prostate Cancer Treated With Novel Hormonal Therapy or Chemotherapy: A Systematic Review and Meta-analysis
Source: Front Oncol. 2020 Nov 30;10:572590. doi: 10.3389/fonc.2020.572590 (PMC7793884; doi:10.3389/fonc.2020.572590)
Supplement: Supplementary file 1 [file DataSheet_1.docx]

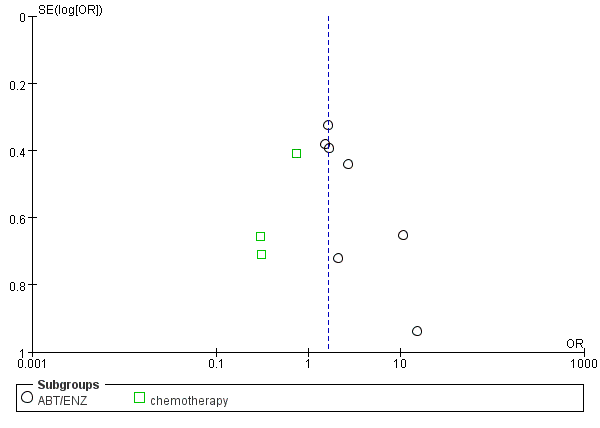


**Figure S1 – the funnel plot for meta-analysis of the** **AR-V7 positive proportion** **conversion after treatment.**

**
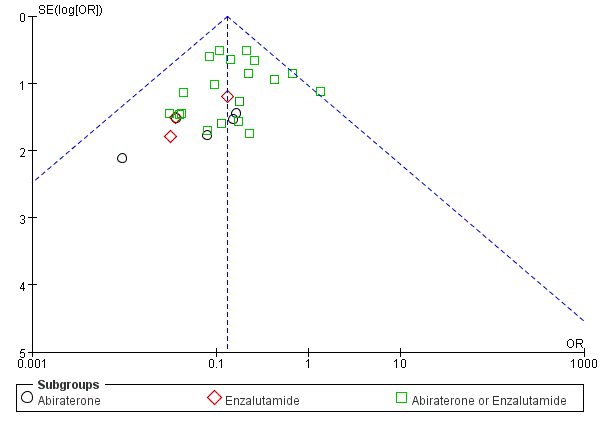
**

**Figure S2 – the funnel plot for meta-analysis of PSA response rate after NHT with different AR-V7 status.**

**
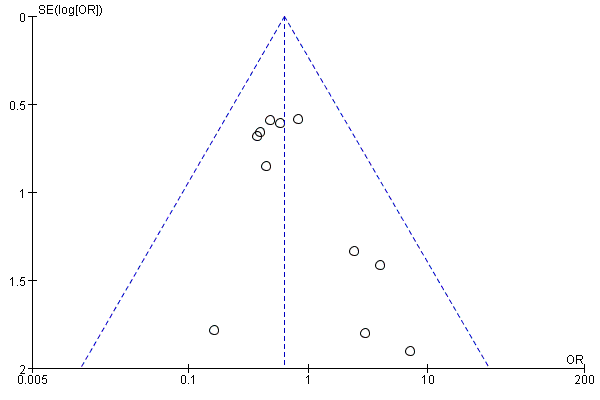
**

**Figure S3 – the funnel plot for meta-analysis of PSA response rate after chemotherapy with different AR-V7 status.**


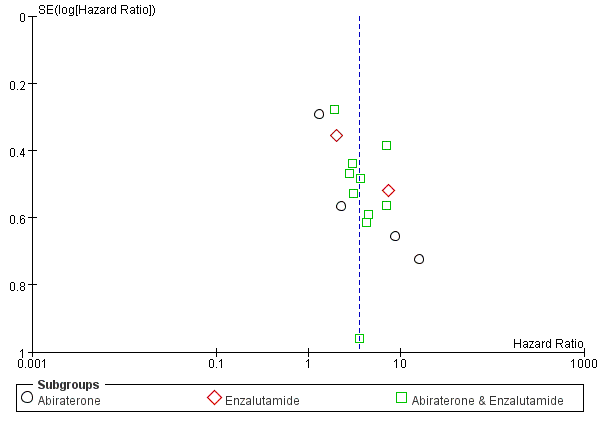


**Figure S4 A– the funnel plot for meta-analysis of the progression free survival of NHT in CRPC patients with different AR-V7 status.**


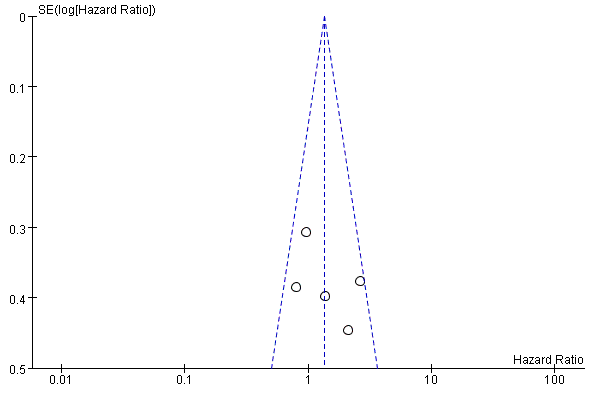


**Figure S4 B– the funnel plot for meta-analysis of the progression free survival of chemotherapy in CRPC patients with different AR-V7 status.**

**
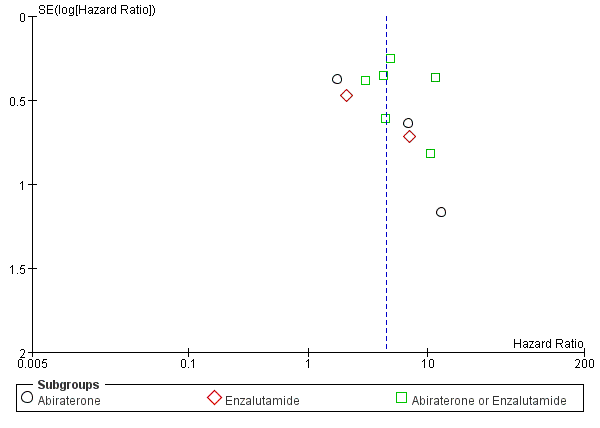
**

**Figure S5A – the funnel plot for meta-analysis of the overall survival in CRPC patients received NHT with different AR-V7 status.**

**
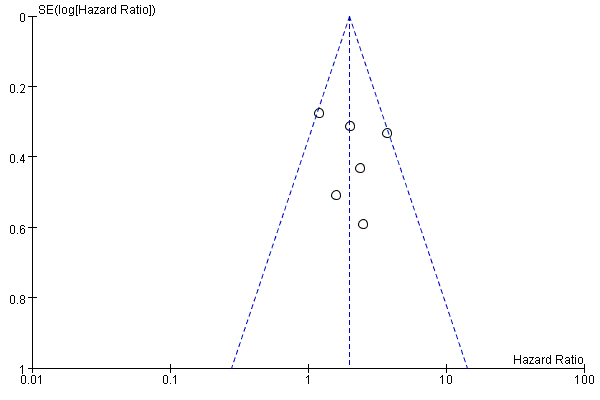
**

**Figure S5B – the funnel plot for meta-analysis of the overall survival in CRPC patients received chemotherapy with different AR-V7 status.**

**
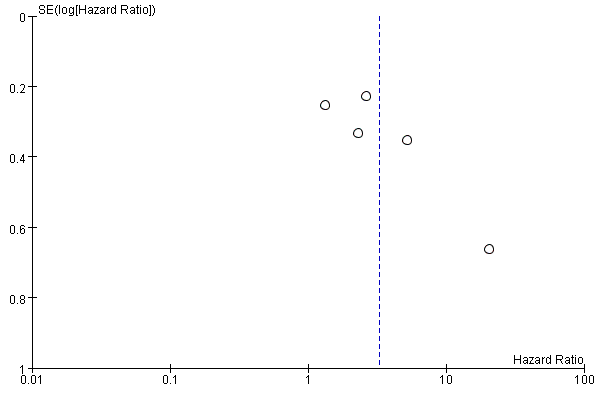
**

**Figure S5C – the funnel plot for meta-analysis of the overall survival in CRPC patients received unselected heterogeneity with different AR-V7 status.**

**Table S1 – Target specimens and AR-V7 detection assay of studies in the change of AR-V7-positive proportion after treatment meta-analysis.**

| Study | Target specimens | AR-V7 detection assay |
| --- | --- | --- |
| Antonarakis[[1](#_ENREF_1)] | CTC mRNA | CTC analyses were conducted using the commercially-available AlereTM CTC AdnaTest  platform (AdnaGen, Langenhagen, Germany) |
| Antonarakis[[2](#_ENREF_2)] | CTC mRNA | CTC analyses were conducted using a modified AdnaTest platform (QIAGEN, Hannover, Germany) |
| Armstrong[[3](#_ENREF_3)] | CTC mRNA | CTCs were analyzed in two central laboratories, the Epic Sciences (Epic; San Diego, CA) and The Johns Hopkins University (JHU; Baltimore, MD) modifiedAdnaTest CTC AR-V7 mRNA assay. |
| Nakazawa[[4](#_ENREF_4)] | CTC mRNA | AdnaTest platform for CTC analysis. CTCs were enriched from peripheral blood using the ProstateCancerSelect kit, mRNA expression analysis was conducted using the ProstateCancerDetect kit. |
| Sharp[[5](#_ENREF_5)] | IHC on FFPE PC patient tissue biopsies | A recombinant rabbit monoclonal antibody(Clone RM7) was developed against CE3 of AR-V7. Patients baseline characteristics and clinical outcomes were compared by positive(nuclear AR-V7 HS > 10) or negative (nuclear AR-V7 HS ≤ 10) AR-V7 status |
| Welti [[6](#_ENREF_6)] | FFPE matched archival and CRPC tissue | Immunohistochemistry.  A rabbit monoclonal antibody (EP343) detected AR-V7, but not AR-FL or ARV567es. |
| Zadra[[7](#_ENREF_7)] | immunofluorescence staining of mCRPC tissue | The slides were incubated with antibodies against ARV7 (Clone RM7, 31-1109-00, RevMAb Biosciences, dilution 1: 300) |
| Antonarakis[[8](#_ENREF_8)] | CTC mRNA | The CTC analyses were conducted using a modification of the commercially available AdnaTest platform (Qiagen) |
| Sieuwerts[[9](#_ENREF_9)] | CTC mRNA | CellSearch-enriched CTCs were subjected to RNA isolation using the AllPrep DNA/RNA Micro Kit (Qiagen, Hilden, Germany) |

FFPE=formalin fixed paraffin embedded tissue, RRP= radical prostatectomy, TURP= transurethral resection of prostate, RNA ISH = RNA *in situ* hybridization, qRT-PCR= Quantitative real-time polymerase chain reaction, CTC = circulating tumor cell

**Table S2 –Definition of PSA response, PFS and OS in the studies included in the meta-analysis of prognosis for hormonal therapy and chemotherapy in different AR-V7 states**

| study | PSA response | Progression free survival | Overall survival |
| --- | --- | --- | --- |
| Antonarakis [[1](#_ENREF_1)] | ≥50% decline in PSA level from baseline, maintained for ≥4 weeks at any time after the initiation of therapy | increase in the PSA level of 25% or more above the nadir (and by ≥2 ng per milliliter), with confirmation 4 or more weeks later; worsening disease-related symptoms or new cancer related complications; ≥20% increase in the sum of the diameters of soft-tissue target lesions onCT scanning; ≥2 new bone lesions on bone scanning or death, whichever occurred first. using the PCWG2 criteria | the time to death from any cause |
| Antonarakis[[8](#_ENREF_8)] | ≥50% decline in PSA level from baseline, maintained for ≥3 weeks at any time after the initiation of therapy | increase in the PSA level of 25% or more above the nadir (and by ≥2 ng per milliliter), with confirmation 3 or more weeks later; worsening disease-related symptoms or new cancer related complications; ≥20% increase in the sum of the diameters of soft-tissue target lesions onCT scanning; ≥2 new bone lesions on bone scanning or death, whichever occurred first. using the PCWG2 criteria | the time to death  from any cause |
| Nakazawa[[4](#_ENREF_4)] | ≥50% PSA  decline at any time on therapy, maintained for ≥4 weeks | - | - |
| Scher[[10](#_ENREF_10)] | For ARS inhibitors, “sensitive” was defined as a 50% or greater decline from baseline at 12 weeks, but for taxane treatment, 12 weeks or more was used because the maximal decline may occur later | A minimum of 2 rising prostate specific antigen (PSA) levels 1 or more weeks apart, new lesions by bone scintigraphy, and/or new or enlarging soft tissue lesions by CT or MRI, using the PCWG2 criteria | the time to death  from any cause |
| Steinestel[[11](#_ENREF_11)] | ≥50% decline  in PSA from baseline maintained for ≥4 weeks at any  time after the initiation of therapy | - | - |
| Todenhofer[[12](#_ENREF_12)] | ≥50% decline  in PSA from baseline maintained for ≥3 weeks at any  time after the initiation of therapy | using the PCWG2 criteria | the time to death  from any cause |
| Welti[[6](#_ENREF_6)] | ≥50% decline in PSA from  baseline at 12 wk | - | The date of CRPC biopsy to the date of last contact or date of death from any cause |
| Onstenk[[13](#_ENREF_13)] | 30% or 50% decline in PSA level from baseline to 12 wk or earlier in the case of  treatment discontinuation | using the PCWG2 criteria | interval between registration and death |
| Qu F[[14](#_ENREF_14)] | - | time from treatment initiation until the date of drug discontinuation for any reason, or censored at the date of last follow-up for patients who were still on therapy | the time to death from any cause |
| Antonarakis[[2](#_ENREF_2)] | PSA response was defined as the proportion of patients with a ≥50% PSA decline from baseline at any time point after therapy (and maintained for ≥3 weeks) | progression was defined as symptomatic progression (worsening disease-related symptoms or new cancer-related complications) or radiologic progression (on CT scan: ≥20% enlargement in sum diameter of target lesions [Response Evaluation Criteria in Solid Tumors];on bone scan: two or more new bone lesions not caused by flare), or death, whichever occurred first | The interval from enrolment to death from any cause |
| Del Re [[15](#_ENREF_15)] | ≥50% reduction in PSA level from baseline |  |  |
| Zhu, Y.[[16](#_ENREF_16)] | using the PCWG2 criteria | Clinical/radiographic progression was defined as symptomatic progression or radiographic progression according to modified PCWG2 criteria and the RECIST 1.1 criteria on CT scans. |  |
| To SQ.[[17](#_ENREF_17)] | PSA response rates (defined as PSA decrease 50%, confirmed 3 wk later) | PSA progression-free survival (PSA-PFS; PCWG3 criteria) |  |
| Seitz AK.[[18](#_ENREF_18)] | ≥50% reduction in PSA level from baseline | PCWG2 criteria, Response Evaluation Criteria In Solid Tumors | PCWG2 criteria |
| De Laere, B. [[19](#_ENREF_19)] | ≥50% reduction in PSA level from baseline | time to no longer of clinical benefit (NLCB) |  |
| Scher H.I.[[20](#_ENREF_20)] | ≥50% PSA decline at 12 weeks |  | from initiation of therapy to death from any cause |
| Takeuchi T.[[21](#_ENREF_21)] | ≥50% reduction in PSA level from baseline |  |  |
| Okegawa [[22](#_ENREF_22)] | 50% or greater decline from baseline at 12 weeks | using the PCWG2 criteria | from initiation of therapy to death from any cause |
| Kohli M.[[23](#_ENREF_23)] | using the PCWG2 criteria | using the PCWG2 criteria |  |
| Tommasi S.[[24](#_ENREF_24)] | PSA reduction ≥50% from the baseline | Not mentioned | from initiation of therapy to death from any cause |
| Maillet[[25](#_ENREF_25)] | A decline of >50% from baseline. | PSA progression and Radiological progression was defined using PCWG3 criteria | PSA or radiological progression-free survival (PFS) and overall survival were assessed. |
| Armstrong[[3](#_ENREF_3)] | 50% or greater PSA declines | defined from date of registration to clinical/radiographic progression or death, whichever occurred first. |  |
| Cattrini[[26](#_ENREF_26)] | a decline of at least 50% in PSA values from treatment start. |  | the time elapsed from blood  collection date and the date of death for any cause. |
| Chung[[27](#_ENREF_27)] | best PSA response (maximum percentage decrease in PSA level from baseline) | PSA progression and Radiological progression was defined using PCWG3 criteria | Secondary outcomes included overall survival |
| Del Re[[28](#_ENREF_28)] | consistent with the Prostate Cancer Working Group-2 guidelines | consistent with the Prostate Cancer Working Group-2 guidelines |  |
| El-Heliebi[[29](#_ENREF_29)] | overview of PSA response is shown in a waterfall plot |  |  |
| Sharp[[5](#_ENREF_5)] | 50% PSA fall | Time to PSA progression was defined as time from start of therapy to first PSA increase that is ≥ 25% and ≥ 2µg/L above the PSA nadir. Time to clinical/radiological progression was defined as time from start of therapy to documented radiological progression or clinical progression | Overall survival was defined as time from start of therapy to date of death or last follow up/contact. |
| Sieuwerts[[9](#_ENREF_9)] | 30% and/or 50% decline from baseline to 12 weeks |  | from the registration date until the date of death or last contact in the case of baseline parameters |
| Tagawa[[30](#_ENREF_30)] | At least a 50% PSA reduction from baseline either by C5D1 (prior to switch) or at any point during the treatment | PFS was defined between randomization and the first documentation of radiographic tumor progression (RECIST 1.1), clinical progression, PSA progression, or death from any cause. | |
| Worroll[[31](#_ENREF_31)] | ≥50% decline in 12-week | using PCWG3 criteria |  |
| Belderbos[[32](#_ENREF_32)] |  |  | OS was calculated from the date when blood samples were withdrawn until the date of death from any cause or end of the study, whichever came first |
| Sharp[[33](#_ENREF_33)] | - | - | overall survival was defined as time from PB draw to date of death or last follow up/contact. |
| Graf[[34](#_ENREF_34)] | - | OS was calculated from the time of treatment decision to death. Patients alive at last follow-up were right-censored. | |
| Erb [[35](#_ENREF_35)] | PSA response rates (≥50%) to treatment | Time-to-event outcomes (biochemical relapse and radiological relapse) were evaluated | |
| Kwan [[36](#_ENREF_36)] | PSA decline from baseline of 50%, confirmed 3 weeks later | using PCWG3 criteria | |

IQR= inter quartile range; SD= standard deviation; ALP = alkaline phosphatase; AR-V7 = androgen receptor splice variant 7; CTC = circulating tumor cell; PSA = prostate-specific antigen; PCWG2=Prostate Cancer Working Group 2

1. Antonarakis ES, Lu C, Wang H, Luber B, Nakazawa M, Roeser JC et al. AR-V7 and resistance to enzalutamide and abiraterone in prostate cancer. The New England journal of medicine. 2014;371(11):1028-38. doi:10.1056/NEJMoa1315815.

2. Antonarakis ES, Lu C, Luber B, Wang H, Chen Y, Zhu Y et al. Clinical Significance of Androgen Receptor Splice Variant-7 mRNA Detection in Circulating Tumor Cells of Men With Metastatic Castration-Resistant Prostate Cancer Treated With First- and Second-Line Abiraterone and Enzalutamide. Journal of clinical oncology : official journal of the American Society of Clinical Oncology. 2017:JCO2016701961. doi:10.1200/jco.2016.70.1961.

3. Armstrong AJ, Halabi S, Luo J, Nanus DM, Giannakakou P, Szmulewitz RZ et al. Prospective Multicenter Validation of Androgen Receptor Splice Variant 7 and Hormone Therapy Resistance in High-Risk Castration-Resistant Prostate Cancer: The PROPHECY Study. Journal of clinical oncology : official journal of the American Society of Clinical Oncology. 2019;37(13):1120-9. doi:10.1200/jco.18.01731.

4. Nakazawa M, Lu C, Chen Y, Paller CJ, Carducci MA, Eisenberger MA et al. Serial blood-based analysis of AR-V7 in men with advanced prostate cancer. Annals of oncology : official journal of the European Society for Medical Oncology / ESMO. 2015;26(9):1859-65. doi:10.1093/annonc/mdv282.

5. Sharp A, Coleman I, Yuan W, Sprenger C, Dolling D, Rodrigues DN et al. Androgen receptor splice variant-7 expression emerges with castration resistance in prostate cancer. The Journal of clinical investigation. 2019;129(1):192-208. doi:10.1172/jci122819.

6. Welti J, Rodrigues DN, Sharp A, Sun S, Lorente D, Riisnaes R et al. Analytical Validation and Clinical Qualification of a New Immunohistochemical Assay for Androgen Receptor Splice Variant-7 Protein Expression in Metastatic Castration-resistant Prostate Cancer. European urology. 2016. doi:10.1016/j.eururo.2016.03.049.

7. Zadra G, Ribeiro CF, Chetta P, Ho Y, Cacciatore S, Gao X et al. Inhibition of de novo lipogenesis targets androgen receptor signaling in castration-resistant prostate cancer. Proceedings of the National Academy of Sciences of the United States of America. 2019;116(2):631-40. doi:10.1073/pnas.1808834116.

8. Antonarakis ES, Lu C, Luber B, Wang H, Chen Y, Nakazawa M et al. Androgen Receptor Splice Variant 7 and Efficacy of Taxane Chemotherapy in Patients With Metastatic Castration-Resistant Prostate Cancer. JAMA oncology. 2015;1(5):582-91. doi:10.1001/jamaoncol.2015.1341.

9. Sieuwerts AM, Onstenk W, Kraan J, Beaufort CM, Van M, De Laere B et al. AR splice variants in circulating tumor cells of patients with castration-resistant prostate cancer: relation with outcome to cabazitaxel. Mol Oncol. 2019;13(8):1795-807. doi:10.1002/1878-0261.12529.

10. Scher HI, Lu D, Schreiber NA, Louw J, Graf RP, Vargas HA et al. Association of AR-V7 on Circulating Tumor Cells as a Treatment-Specific Biomarker With Outcomes and Survival in Castration-Resistant Prostate Cancer. JAMA oncology. 2016. doi:10.1001/jamaoncol.2016.1828.

11. Steinestel J, Luedeke M, Arndt A, Schnoeller TJ, Lennerz JK, Wurm C et al. Todenhofer T,Detecting predictive androgen receptor modifications in circulating prostate cancer cells. Oncotarget. 2015. doi:10.18632/oncotarget.3925.

12. Todenhofer T, Azad A, Stewart C, Gao J, Eigl BJ, Gleave ME et al. AR-V7 transcripts in whole blood RNA of patients with metastatic castration resistant prostate cancer correlate with response to Abiraterone acetate. The Journal of urology. 2016. doi:10.1016/j.juro.2016.06.094.

13. Onstenk W, Sieuwerts AM, Kraan J, Van M, Nieuweboer AJ, Mathijssen RH et al. Efficacy of Cabazitaxel in Castration-resistant Prostate Cancer Is Independent of the Presence of AR-V7 in Circulating Tumor Cells. European urology. 2015;68(6):939-45. doi:10.1016/j.eururo.2015.07.007.

14. Qu F, Xie W, Nakabayashi M, Zhang H, Jeong SH, Wang X et al. Association of AR-V7 and prostate specific antigen RNA levels in blood with efficacy of abiraterone acetate and enzalutamide treatment in men with prostate cancer. Clinical cancer research : an official journal of the American Association for Cancer Research. 2016. doi:10.1158/1078-0432.ccr-16-1070.

15. Del Re M, Biasco E, Crucitta S, Derosa L, Rofi E, Orlandini C et al. The Detection of Androgen Receptor Splice Variant 7 in Plasma-derived Exosomal RNA Strongly Predicts Resistance to Hormonal Therapy in Metastatic Prostate Cancer Patients. European urology. 2017;71(4):680-7. doi:10.1016/j.eururo.2016.08.012.

16. Zhu Y, Sharp A, Anderson CM, Silberstein JL, Taylor M, Lu C et al. Novel Junction-specific and Quantifiable In Situ Detection of AR-V7 and its Clinical Correlates in Metastatic Castration-resistant Prostate Cancer. European urology. 2018;73(5):727-35. doi:10.1016/j.eururo.2017.08.009.

17. To SQ, Kwan EM, Fettke HC, Mant A, Docanto MM, Martelotto L et al. Expression of Androgen Receptor Splice Variant 7 or 9 in Whole Blood Does Not Predict Response to Androgen-Axis-targeting Agents in Metastatic Castration-resistant Prostate Cancer. European urology. 2018. doi:10.1016/j.eururo.2018.01.007.

18. Seitz AK, Thoene S, Bietenbeck A, Nawroth R, Tauber R, Thalgott M et al. AR-V7 in Peripheral Whole Blood of Patients with Castration-resistant Prostate Cancer: Association with Treatment-specific Outcome Under Abiraterone and Enzalutamide. European urology. 2017. doi:10.1016/j.eururo.2017.07.024.

19. De Laere B, van Dam PJ, Whitington T, Mayrhofer M, Diaz EH, Van den Eynden G et al. Comprehensive Profiling of the Androgen Receptor in Liquid Biopsies from Castration-resistant Prostate Cancer Reveals Novel Intra-AR Structural Variation and Splice Variant Expression Patterns. European urology. 2017. doi:10.1016/j.eururo.2017.01.011.

20. Scher HI, Graf RP, Schreiber NA, McLaughlin B, Lu D, Louw J et al. Nuclear-specific AR-V7 Protein Localization is Necessary to Guide Treatment Selection in Metastatic Castration-resistant Prostate Cancer. European urology. 2017;71(6):874-82. doi:10.1016/j.eururo.2016.11.024.

21. Takeuchi T, Okuno Y, Hattori-Kato M, Zaitsu M, Mikami K. Detection of AR-V7 mRNA in whole blood may not predict the effectiveness of novel endocrine drugs for castration-resistant prostate cancer. Research and reports in urology. 2016;8:21-5. doi:10.2147/rru.s98877.

22. Okegawa T, Ninomiya N, Masuda K, Nakamura Y, Tambo M, Nutahara K. AR-V7 in circulating tumor cells cluster as a predictive biomarker of abiraterone acetate and enzalutamide treatment in castration-resistant prostate cancer patients. The Prostate. 2018;78(8):576-82. doi:10.1002/pros.23501.

23. Kohli M, Ho Y, Hillman DW, Van Etten JL, Henzler C, Yang R et al. Androgen Receptor Variant AR-V9 Is Coexpressed with AR-V7 in Prostate Cancer Metastases and Predicts Abiraterone Resistance. Clinical cancer research : an official journal of the American Association for Cancer Research. 2017;23(16):4704-15. doi:10.1158/1078-0432.ccr-17-0017.

24. Tommasi S, Pilato B, Carella C, Lasorella A, Danza K, Vallini I et al. Standardization of CTC AR-V7 PCR assay and evaluation of its role in castration resistant prostate cancer progression. The Prostate. 2018. doi:10.1002/pros.23710.

25. Maillet D, Allioli N, Peron J, Plesa A, Decaussin-Petrucci M, Tartas S et al. Improved Androgen Receptor Splice Variant 7 Detection Using a Highly Sensitive Assay to Predict Resistance to Abiraterone or Enzalutamide in Metastatic Prostate Cancer Patients. Eur Urol Oncol. 2019. doi:10.1016/j.euo.2019.08.010.

26. Cattrini C, Rubagotti A, Zinoli L, Cerbone L, Zanardi E, Capaia M et al. Role of Circulating Tumor Cells (CTC), Androgen Receptor Full Length (AR-FL) and Androgen Receptor Splice Variant 7 (AR-V7) in a Prospective Cohort of Castration-Resistant Metastatic Prostate Cancer Patients. Cancers (Basel). 2019;11(9). doi:10.3390/cancers11091365.

27. Chung JS, Wang Y, Henderson J, Singhal U, Qiao Y, Zaslavsky AB et al. Circulating Tumor Cell-Based Molecular Classifier for Predicting Resistance to Abiraterone and Enzalutamide in Metastatic Castration-Resistant Prostate Cancer. Neoplasia (New York, NY). 2019;21(8):802-9. doi:10.1016/j.neo.2019.06.002.

28. Del Re M, Crucitta S, Sbrana A, Rofi E, Paolieri F, Gianfilippo G et al. AR-V7 and AR-FL expression is associated with clinical outcome: a translational study in patients with castrate resistant prostate cancer. BJU international. 2019. doi:10.1111/bju.14792.

29. El-Heliebi A, Hille C, Laxman N, Svedlund J, Haudum C, Ercan E et al. In Situ Detection and Quantification of AR-V7, AR-FL, PSA, and KRAS Point Mutations in Circulating Tumor Cells. Clin Chem. 2018;64(3):536-46. doi:10.1373/clinchem.2017.281295.

30. Tagawa ST, Antonarakis ES, Gjyrezi A, Galletti G, Kim S, Worroll D et al. Expression of AR-V7 and ARv(567es) in Circulating Tumor Cells Correlates with Outcomes to Taxane Therapy in Men with Metastatic Prostate Cancer Treated in TAXYNERGY. Clinical cancer research : an official journal of the American Association for Cancer Research. 2019;25(6):1880-8. doi:10.1158/1078-0432.ccr-18-0320.

31. Worroll D, Galletti G, Gjyrezi A, Nanus DM, Tagawa ST, Giannakakou P. Androgen receptor nuclear localization correlates with AR-V7 mRNA expression in circulating tumor cells (CTCs) from metastatic castration resistance prostate cancer patients. Phys Biol. 2019;16(3):036003. doi:10.1088/1478-3975/ab073a.

32. Belderbos BPS, Sieuwerts AM, Hoop EO, Mostert B, Kraan J, Hamberg P et al. Associations between AR-V7 status in circulating tumour cells, circulating tumour cell count and survival in men with metastatic castration-resistant prostate cancer. European journal of cancer (Oxford, England : 1990). 2019;121:48-54. doi:10.1016/j.ejca.2019.08.005.

33. Sharp A, Welti JC, Lambros MBK, Dolling D, Rodrigues DN, Pope L et al. Clinical Utility of Circulating Tumour Cell Androgen Receptor Splice Variant-7 Status in Metastatic Castration-resistant Prostate Cancer. European urology. 2019. doi:10.1016/j.eururo.2019.04.006.

34. Graf RP, Hullings M, Barnett ES, Carbone E, Dittamore R, Scher HI. Clinical Utility of the Nuclear-localized AR-V7 Biomarker in Circulating Tumor Cells in Improving Physician Treatment Choice in Castration-resistant Prostate Cancer. European urology. 2019. doi:10.1016/j.eururo.2019.08.020.

35. Erb HHH, Sparwasser P, Diehl T, Hemmerlein-Thomas M, Tsaur I, Jungel E et al. AR-V7 Protein Expression in Circulating Tumour Cells Is Not Predictive of Treatment Response in mCRPC. Urologia internationalis. 2020:1-10. doi:10.1159/000504416.

36. Kwan EM, Fettke H, Docanto MM, To SQ, Bukczynska P, Mant A et al. Prognostic Utility of a Whole-blood Androgen Receptor-based Gene Signature in Metastatic Castration-resistant Prostate Cancer. Eur Urol Focus. 2019. doi:10.1016/j.euf.2019.04.020.
